# A cutting-edge new framework for the pain management in children: nanotechnology

**DOI:** 10.3389/fnmol.2024.1391092

**Published:** 2024-09-10

**Authors:** Iuliana Magdalena Starcea, Ancuta Lupu, Ana Maria Nistor, Maria Adriana Mocanu, Roxana Alexandra Bogos, Alice Azoicai, Diana Cira, Madalina Beldie, Vasile Valeriu Lupu, Ionela Daniela Morariu, Valentin Munteanu, Razvan Tudor Tepordei, Ileana Ioniuc

**Affiliations:** ^1^Pediatrics Department, “Grigore T. Popa” University of Medicine and Pharmacy, Iasi, Romania; ^2^Nephrology Division, St. Mary’s Emergency Children Hospital, Iasi, Romania; ^3^Faculty of Pharmacy, “Grigore T. Popa” University of Medicine and Pharmacy, Iasi, Romania; ^4^Faculty of Medical Bioengineering, “Grigore T. Popa” University of Medicine and Pharmacy, Iasi, Romania; ^5^Faculty of Medicine, “Grigore T. Popa” University of Medicine and Pharmacy, Iasi, Romania

**Keywords:** nanomedicine, pain therapy, children, nanotechnology, nanoparticle

## Abstract

Pain is a subjective concept which is ever-present in the medical field. Health professionals are confronted with a variety of pain types and sources, as well as the challenge of managing a patient with acute or chronic suffering. An even bigger challenge is presented in the pediatric population, which often cannot quantify pain in a numerical scale like adults. Infants and small children especially show their discomfort through behavioral and physiological indicators, leaving the health provider with the task of rating the pain. Depending on the pathophysiology of it, pain can be classified as neuropathic or nociceptive, with the first being defined by an irregular signal processing in the nervous system and the second appearing in cases of direct tissue damage or prolonged contact with a certain stimulant. The approach is generally either pharmacological or non-pharmacological and it can vary from using NSAIDs, local anesthetics, opiates to physical and psychological routes. Unfortunately, some pathologies involve either intense or chronic pain that cannot be managed with traditional methods. Recent studies have involved nanoparticles with special characteristics such as small dimension and large surface area that can facilitate carrying treatments to tissues and even offer intrinsic analgesic properties. Pediatrics has benefited significantly from the application of nanotechnology, which has enabled the development of novel strategies for drug delivery, disease diagnosis, and tissue engineering. This narrative review aims to evaluate the role of nanotechnology in current pain therapy, with emphasis on pain in children.

## 1 Introduction

Pain is a subjective concept which is ever-present in the medical field. Health professionals are confronted with a variety of pain types and sources, as well as the challenge of managing a patient with acute or chronic suffering. The International Association for the Study of Pain (IASP) has recently revised the definition of this concept as “An unpleasant sensory and emotional experience associated with, or resembling that associated with, actual or potential tissue damage” (Raja et al., 2020).

An even bigger challenge is presented in the pediatric population, which often cannot quantify pain in a numerical scale like adults. Pediatric chronic pain impacts a considerable portion of young individuals by it worldwide, ranging from 11 to 38%, with about 3% to 5% experiencing notable levels of disability (Neville et al., 2020). In children’s hospitals, it’s well-established that over 10% of hospitalized children grapple with chronic pain (Friedrichsdorf and Goubert, 2020). Unlike the criteria in adult medicine, defining chronic pain in children doesn’t strictly rely on fixed timeframes (e.g., 3 months). Instead, it’s characterized functionally, as pain persisting beyond the typical healing period, losing the acute warning function of normal pain sensation (Treede et al., 2015).

Pain management in pediatric patients often falls short of optimal levels. Self-reporting is the preferred method when feasible, given the subjective nature of pain. However, in cases where infants, small children, or those unable to communicate accurately, alternative assessment methods like behavior-based measures can supplement or substitute self-reporting to ensure effective pain evaluation (Gai et al., 2020).

**FIGURE 1:**
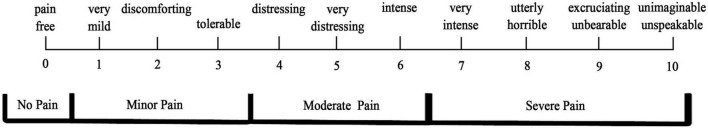
Numerical Rating Scale used for older children.

Infants and small children especially show their discomfort through behavioral and physiological indicators, leaving the health provider with the task of rating the pain. Starting at about three years old, kids can comfortably use scales for improving the accuracy of the assessment. There are a number of options in regards with pain measurement tools. Self-report scales such as Numerical Rating Scale (NRS) is helpful with children above eight years old and they are asked to rate their pain from no pain to the worst possible pain by putting a mark on the scale where they rate the hurt (Figure 1; He et al., 2019).

**FIGURE 2:**
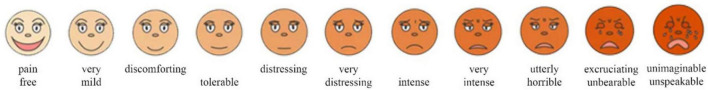
Faces Pain Scale Revised for smaller children.

For smaller children or ones that cannot accurately point to a number on the NRS, the Faces Pain Scale Revised (FPS-R) can pe utilized. Using a series of six cartoon faces ranging from neutral to very distressed, the patient is asked which figure best represents the amount of pain they are in (Figure 2; Arnold et al., 2023).

For even smaller patients or the ones with development disabilities, the Faces Legs Activity Cry Consolability Revised Scale (FLACC-R) was created. It relies on behavioral indicators and it consists of a checklist that the medical professional can use to objectively asses pain (Table 1). In patients who are awake they need to be observed for 2–5 min, while asleep children need to be assessed for 5 min or longer. The sum of the score varies from 0 = relaxed and comfortable, 1–3 = mild discomfort, 4–6 = moderate pain to 7–10 = severe discomfort/pain (Papa et al., 2023).

**TABLE 1:** Face Legs Activity Cry Consolability Revised Scale.

Face		
0−No particular expression or smile	1−Occasional grimace or frown, withdrawn, disinterested	2−Frequent to constant frown, clenched jaw, quivering chin
**Legs**		
0−Normal position or relaxed	1−Uneasy, restless, tense	2−Kicking or legs drawn up
**Activity**		
0−Lying quietly, normal position, moves easily	1−Squirming, shifting back/forth, tense	2−Arched, rigid, or jerking
**Cry**		
0−No cry, awake or asleep	1−Moans or whimpers, occasional complaint	2−Crying steadily, screams or sobs, frequent complaints
**Consolability**		
0−Content, relaxed	1−Reassured by occasional touching, hugging, or “talking to,” distractible	2−Difficult to console or comfort

Finally, for infants and newborns, the Neonatal/Infant Pain Scale (NIPS) is used. Recommended for patients less than 1-year-old, this tool utilizes behavioral patterns such as crying, breathing or facial expressions to assess discomfort. A score greater than 3 indicates pain (Table 2; Mondardini et al., 2023).

**TABLE 2:** Neonatal/Infant Pain Scale.

Facial expression	
0 – Relaxed muscles	Restful face, neutral expression
1 – Grimace	Tight facial muscles, furrowed brow, chin, jaw, negative facial expression - nose, mouth and brow
**Cry**	
0 – No cry	Quiet, not crying
1 – Whimper	Mild moaning, intermittent
2 – Vigorous Cry	Loud scream, rising, shrill, continuous (note: silent cry may be scored if baby is intubated as evidenced by obvious mouth and facial movement)
**Breathing patterns**	
0 – Relaxed	Usual pattern for this infant
1 – Change in breathing	Indrawing, irregular, faster than usual, gagging, breath holding
**Arms**	
0 – Relaxed/Restrained	No muscular rigidity, occasional random movements
1 – Flexed/Extended	Tense, straight legs, rigid/or rapid extension, flexion
**Legs**	
0 – Relaxed/Restrained	No muscular rigidity, occasional random movements
1 – Flexed/Extended	Tense, straight legs, rigid/or rapid extension, flexion
**State of arousal**	
0 – Sleeping/Awake	Quiet, peaceful, sleeping or alert, random leg movements
1 – Fussy	Alert, restless and thrashing

Nanomedicine holds the potential to enhance the efficiency, as well as the accuracy, of diagnosis and various targeted therapies. The unique physicochemical and biological properties of nanomaterials, such as high surface-to-volume ratios and distinctive structural properties, enable surface modification, the ability to penetrate biological barriers, and extended blood circulation time, rendering them suitable for both diagnostic and therapeutic applications. Recent research has also focused on the development of non-invasive nanodevices for genomic analysis and molecular diagnosis, as well as their utilization in various therapies (Bidve et al., 2020).

Nanomedicine, a burgeoning field in medicine, harnesses nanotechnology to revolutionize disease diagnosis, treatment, and prevention. One key benefit lies in its capability to precisely deliver drugs to disease sites, reducing adverse effects and enhancing efficacy. Additionally, nanomedicine’s adeptness at bypassing biological barriers, such as the blood-brain barrier, extends its utility by ensuring drugs reach their targets unhindered. Moreover, the integration of nanoscale materials in regenerative medicine holds promise for tissue regeneration and repair (Soliman, 2023).

However, nanomedicine is not without its challenges. Concerns revolve around nanoparticle toxicity, the complexities of large-scale nanoparticle manufacturing, and the imperative task of guaranteeing their safety and efficacy. These hurdles underscore the necessity for meticulous research and development efforts to realize the full potential of nanomedicine while mitigating associated risks (Chen et al., 2018).

Pediatrics has benefited significantly from the application of nanotechnology, which has enabled the development of novel strategies for drug delivery, disease diagnosis, and tissue engineering (Omidian and Mfoafo, 2023). By manipulating materials at the nanoscale, nanotechnology has the potential to increase the efficacy of drugs and decrease their toxicity.

We performed a narrative literature review using the latest articles on this topic, with the aim of proving the important role of nanotechnology in the actual pain treatment of children.

## 2 Pain management in children

Pain is a multidimensional phenomenon involving sensory, emotional, motivational, environmental, and cognitive components, with many peculiarities in the child. Initiation of analgesic therapy should take into account certain particularities of pharmacokinetics and pharmacodynamics, as the liver enzyme system involved in the metabolism of certain drugs matures with age, protein binding capacity is low, and the clearance of the analgesics is different between age groups (Goţia and Russu, 2022). The choice of analgesic treatment should be individualized for the child in order to obtain an optimal ratio between analgesia and side effects. WHO suggests starting pain treatment according to severity: mild pain (step I): non-opioid analgesic agents prostaglandin synthetase inhibitor (PGSIs); in moderate pain (step II): weak opioid painkillers or combinations between a weak PGSIs and opioid (e.g., paracetamol or a nonsteroidal anti-inflammatory NSAID + codeine); and in important, severe pain (step III): strong opioids (with administration, mainly parenterally); in each step, according to need and experience, various adjuvant therapies can be combined. Adjuvant therapies encompasses a diverse array of medications spanning various classes. While initially prescribed for purposes beyond pain management, these drugs prove invaluable in alleviating various painful conditions (Anekar et al., 2024).

Secondary analgesics are characterized by their primary clinical activity, with pain relief being a secondary effect. They can effectively serve as standalone analgesic agents for treating a range of pain conditions (Queremel Milani and Davis, 2024). In chronic pain, important, very severe, and sometimes specific methods of anesthesia are required (e.g., neurolytic nerve blockages, intrathecal anesthetics, etc.) (Smith et al., 2022).

### 2.1 Mild pain (step I) - non-opioid analgesics

PGSIs interfere with the function of cyclooxygenases, preventing the conversion of arachidonic acid into prostaglandins and thromboxanes, which are involved in the production of hyperemia, edema, and pain in the damaged tissues. Prostaglandins and thromboxanes combine their effects with other inflammation mediators (bradykinins, leukotrienes, serotonin, histamine) in stimulating free nerve endings, followed by stimulation of the central nervous (Chung et al., 2018).

Examples of PGSIs are:

1. paraaminophenolic derivatives (acetaminophen, phenacetin),
2. salicylates (salicylic acetylic acid) extremely rarely used in pediatric pain
3. NSAIDs (ibuprofen, naproxen, tolmetin, ketorolac).

#### 2.1.1 Paraaminofenolic derivatives

Phenacetine−is not used due to the fact that the analgesic activity is given by the rapid biotransformation into acetaminophen, and its administration is accompanied by much more adverse reactions (methemoglobinemia, hemolysis, nephritis) than in the case of its use (Schipton, 2000).

Acetaminophen is the antipyretic most used in childhood, and has, at the same serum concentrations, important analgesic and mild anti-inflammatory action. It is evenly dis-tributed throughout the human body. It is a substance that is rapidly and almost completely absorbed in the intestine (maximum serum concentration is reached at 30–60 min); it is metabolized in the liver (sulphoconjugation, glycuronoconjugeration, oxidation by cytochrome P450) and secreted in the urine (3% in free form). It acts centrally, the duration of action is 4–6 h. Has a limited anti-inflammatory effect (Schipton, 2000; Wang et al., 2019). Compared to NSAIDs, acetaminophen has fewer side effects: it does not cause irritation or digestive ulcers and does not inhibit platelet function. The most feared complication is liver damage, which can go up to acute fulminant liver failure (the antidote is N acetyl-cysteine); young children are more resistant to hepatotoxicity of paracetamol, due to metabolic differences−predominant sulphoconjugation. Contraindications are significant impairment of liver and/or renal function and deficiency of glucose6-phosphate dehydrogenase (G6PD) (Wang et al., 2019; Mach et al., 2014). As far as drug interactions are concerned, it may be used simultaneously with high-grade painkillers, sometimes even allowing for the reduction of morphine doses.

#### 2.1.2 Non-steroidal anti-inflammatory drugs (NSAIDs)

They have extremely widespread use in pediatrics fever, acute or chronic pain, or combinations, in order to reduce opioid analgesic doses. The main indication is inflammatory pain. The onset of the analgesic effect is relatively rapid but the anti-inflammatory effect is late and sometimes involves increasing doses for an optimal response (Kela et al., 2021). NSAIDs act both by inactivating the chemical action of cyclooxygenases (COX), blocking the production of PG and thromboxanes as well as by inhibiting phosphodiesterases and increasing intra-cellular cyclic AMP (cAMP) (Kela et al., 2021). The classic ones (ibuprofen, diclofenac, indomethacin, aspirin) inhibit both types of Cyclooxygenases, hence the increased frequency of adverse effects and the new non-steroidal anti-inflammatory agents (celecoxib, parecoxib) selectively inhibit cyclooxygenase 2 having analgesic, anti-inflammatory and antipyretic effects (Mach et al., 2014). In a higher degree of pain severity, various combinations of paracetamol or NSAIDs are recommended, especially with weak opioids (codeine, oxycodone) demonstrating an anti-allergic effect superior to doubling the doses of ISPs (Zhuang et al., 2022). Side effects of NSAIDs are important, especially in long-term therapy, and involve the gastrointestinal system, nervous, system, kidney, hematological system, and liver. NSAIDs can cause bronchoconstriction in asthmatics, or some children with allergic rhinitis and nasal polyposis. These side effects limit their use in patients at risk: major dehydration syndromes, renal failure, allergic diseases, stomach ulcers, and intestinal pathology (Rech et al., 2022). Liver and renal function must be monitored at least at 6-month intervals in children on long-term NSAID treatment (Mach et al., 2014). Rarely have been described skin side effects−from urticaria, or simple itching, to multiform erythema, or the appearance of pseudoporphyria (in children with prolonged treatment with naproxen), central nervous system side effects – headaches, fatigue, agitation, sleep disorders. Ibuprofen has been involved in the development of cases of aseptic meningitis in patients with systemic lupus erythematosus (Schipton, 2000).

Salicylates, derived from salicylic acid, are a subclass of NSAIDs. The utilization of salicylates in childhood has significantly diminished following the documentation of the correlation between aspirin and the development of Reye’s syndrome or metabolic acidosis in newborns. Consequently, its application has been restricted in specific rheumatological conditions or as an antiplatelet medication (Mach et al., 2014). Salicylates reduce pain and swelling by stopping the production of PG and thromboxanes (by acetylating and deactivating cyclooxygenases) in both brain and body tissues (Ailioaie and Ailioaie, 2008).

### 2.2 Moderate pain (step II)−Weak opioids (hydrocodone, codeine, tramadol) with or without non-opioid analgesics and with or without adjuvants

For moderate pain or when initial pain relief methods are ineffective, it is recommended to use weak opioids or combinations of acetaminophen, aspirin, or NSAIDs with a weak, fast-acting opioid like codeine, hydrocodone, tramadol, or propoxyphene. Alternatively, a low dose of a strong opioid like morphine or oxycodone can be used. Additional adjunct medications can also be given to personalize pain control based on individual requirements (Ruoff, 2022; Barnett et al., 2016). Opioids are the primary category of medications used for pain control in children, exhibiting a reduced likelihood of addiction in comparison to adults. Weak opioids such as codeine, oxycodone, and hydrocodone are commonly prescribed to children for the management of moderate pain when the pain relief achieved through the use of non-opioid pain medications is inadequate (Cassidy et al., 2021). The side effects of mild opioids primarily manifest as central respiratory depression, nausea and vomiting, impaired liver and kidney function, delayed menstrual cycle, and notably, constipation (which requires management and prevention) and pruritus.

#### 2.2.1 Codeine

It is a direct agonist of opioid receptors, produces analgesic, antitussive, and respiratory depressive effects, which are comparable to those of morphine. The initial hepatic metabolism enhances the bioavailability and efficacy of codeine as approximately 10% of it is converted into morphine by demethylation. However, individuals with a congenital deficit of the liver enzymes responsible for this transformation, which is very prevalent in the general population, experience a diminished analgesic benefit from codeine (Schipton, 2000). Gastrointestinal or neurological side effects are significant and can occasionally render the use of codeine unbearable. The utilization of codeine in conjunction with acetaminophen has been incorporated into current medical practice and has demonstrated its unequivocal superiority in treating moderate pain. The recommended oral dosage of codeine in combination is 0.5–1 mg/kg administered every 3–4 h (Barnett et al., 2016).

#### 2.2.2 Tramadol

It is the preferred analgesic for the second step of the WHO pain scale. It works by blocking the reception of norepinephrine and serotonin neurotransmitters in the central nervous system. Tramadol is indicated for children over 9 years old, who weigh more than 35 Kg and are experiencing moderate pain (Goţia and Russu, 2022; Smith et al., 2022). Tramadol is an unconventional pain reliever that has effects on the noradrenaline and serotonin systems. Its active metabolite is a mild opioid. There are various medication interactions associated with this chemical, and it is important to consider them. For instance, tramadol can cause convulsions when taken alone, but the risk of convulsions significantly rises when tramadol is taken in combination with certain kinds of antidepressants (Blanco-Nistal and Fernández-Fernández, 2023).

### 2.3 Severe pain (step III)−potent opioids

Here are included morphine, methadone, fentanyl, oxycodone, buprenorphine, tapentadol, hydromorphone, oxymorphone with or without non-opioid analgesics, and with or without adjuvants (Treede et al., 2015). There are no studies on the pediatric use of all known opioid substances.

### 2.4 Secondary analgesics

The pharmacological agents with secondary analgesic activity, currently extensively used in pediatrics, that can be added at any stage of pain in children, are represented by: psychoactive agents (antidepressants, anticonvulsants, anxiolytics), drugs with cardiovascular action, N-methyl-D-aspartate (NMDA) receptor blockers (ketamine, dextromethorphan), muscle relaxants (carisoprodol, chlorzoxazone), enzyme inhibitors, cytotoxic agents, hormones, bisphosphonates, calcitonin, catecholamine precursors, catecholamine antagonists, anesthetics (Tartau and Ionescu, 2022).

#### 2.4.1 Local anesthetics

Lidocaine, bupivacaine, prilocaine, mepivacaine, procaine, cocaine and benzocaine are commonly used in pediatric patients for various applications including topical use, skin infiltration, peripheral nerve blockage, neuraxial epidural blocks, intrathecal infusions, and intravenous infusions. Amides, which are metabolized in the liver, include lidocaine, bupivacaine, prilocaine, and mepivacaine, while esters, which are metabolized in plasma, include procaine, cocaine, and benzocaine. One example of a topical application is eutectic mixture of local anesthetics (EMLA), which is a combination of lidocaine and pyrrocaine. They exhibit rapid efficacy and can enhance both sleep quality and overall patient well-being. Amitriptyline and desipramine, which have been extensively researched, can be prescribed for the treatment of migraine, postherpetic neuralgia, and arthritis. Recent antidepressants, such as selective serotonin reuptake inhibitors like sertraline, have fewer adverse effects yet demonstrate relatively modest efficacy in treating neuropathic pain (Tartau and Ionescu, 2022).

#### 2.4.2 Anticonvulsants

Carbamazepine, phenytoin, sodium valproate, carbamazepine and gabapentin have demonstrated their efficacy in treating neuropathic pain. Among these, gabapentin and carbamazepine have been extensively investigated. The analgesic effect is likely achieved through the reduction of neural excitability. Occasionally, these medications may be recommended together, but often they are delivered sequentially in the therapeutic regimen (Zelter Lonnie and Krane Elliot, 2016).

#### 2.4.3 Steroids

Dexamethasone and prednisone can provide pain relief in conditions including cancer pain, vasculitis (Henoch Schönlein purpura), migraines, or reflex sympathetic dystrophies (Yi et al., 2020; Kwak et al., 2022).

#### 2.4.4 Muscle relaxants

While not actually causing relaxation of the skeletal muscles they are capable of diminishing the perception of pain. They are commonly employed in cases of mild musculoskeletal discomfort. For significant and long-lasting chronic pain, many anesthetic techniques are used, such as regional analgesia, epidural analgesia (for major procedures in the chest or abdomen), peripheral nerve blocks, and intrathecal anesthesia. Thioridazine, haloperidol, chlorpromazine, and olanzapine are major antipsychotics and sedatives that have a contentious use because they can cause significant adverse responses (Ailioaie and Ailioaie, 2008; Inoue et al., 2021).

## 3 Types of nanoparticles

Unfortunately, some pathologies involve either intense or chronic pain that cannot be managed with traditional methods. Uncontrolled drug release is a characteristic of traditional pain management with standard medications, necessitating multiple daily doses to achieve and maintain appropriate plasma concentrations. This erratic delivery leads to fluctuating plasma drug levels, which can sometimes exceed toxic thresholds or fall below effective levels.

Incorporating integrative and complementary non-pharmacological interventions into pediatric chronic pain management has proven to be a viable and effective alternative. These interventions yield long-term benefits by altering the neural circuits that govern habits, emotions, and cognitive responses to pain (Cordeiro Santos et al., 2022; Babaie et al., 2022). The significant disadvantages of available pain control drugs, such as abuse, addiction, tolerance, and the risk of death, have shifted the focus towards developing targeted medications with reduced side effects and prolonged release of active compounds. Integrating pharmacological sciences with nanotechnology has been a crucial step in creating more effective drugs for chronic pain with fewer adverse effects (Chen et al., 2020).

Nanoparticles offer a new view on drug toxicity as well as levels needed for achieving pain control. Since the 1990s, liposomes and polymeric nanoparticles have been utilized to encapsulate opioids, enabling extended-release (ER) formulations and reducing systemic toxicity (Bhansali et al., 2021).

Recent studies have involved nanoparticles with special characteristics such as small dimensions and large surface area that can facilitate carrying treatments to tissues and even offer intrinsic analgesic properties (Lu et al., 2021; Shaban et al., 2019).

Lipid-based nanoparticles have had great success in lowering the dosage of conventional medicine and have been already introduced in clinical aspects, while polymeric nano-particles and carriers like gold, and silica of magnets are being studied for pain modulation (Kaye et al., 2020; An et al., 2020; Qi et al., 2020; Husain et al., 2019).

Lastly, a new type of nanoparticle called theranostics has the potential to provide both diagnosis and pain treatment, making them potentially invaluable for future medicine. The consensus is that particles between 10 and 100 nm are best suited for treatment delivery, anything above 200 nm accumulating in the liver and spleen and particles smaller than 5 nm being eliminated through the urinary system (Santin et al., 2023; Xu et al., 2023; Bhansali et al., 2021). Nanocarriers enhance the delivery of therapeutic agents while minimizing adverse effects. Consequently, recent developments in innovative nanocarriers like lipid-based and polymeric nanoparticles promise significant improvements in pain management quality (Babaie et al., 2022).

**FIGURE 3:**
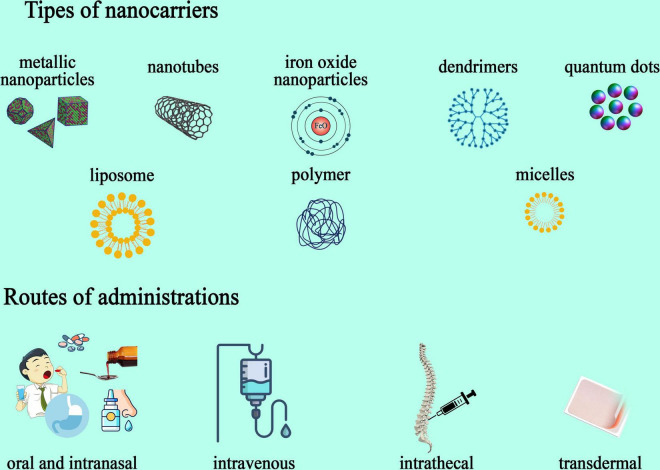
Types of nanoparticles used in drug delivery and route of administration.

Various types of nanoparticles have been developed as carriers and diagnosing agents. According to their material they are classified as (Figure 3).

Inorganic nanoparticles are metallic nanoparticles frequently utilized for delivering drugs and as imaging vehicles. Their high stability and reactivity, as well as photothermic properties, offer useful assets for carrying medicine (López-Rubio et al., 2019). Inorganic particle like Zinc oxide (ZnO), magnesium oxide (MgO), manganese dioxide (MnO2), and magnetite (Fe3O4) have been described in relation to this concept, acting on the NMDA receptors, dopamine receptors, or macrophage activity and blocking pain perception altogether. These novel particles have the ability to deliver anesthetics, opioids, or NSAIDs with much bigger precision and lower levels (Zhang S. et al., 2023). Altering the surface of nanomaterials provides better absorption capacity in specific sites and can further improve drug delivery.

Liposomal nanoparticles - Dendrimers, micelles, liposomes: especially useful for hydrophobic molecules, these particles enhance solubility and prolong the half-life of certain therapeutic agents. Built with hydrophilic components, they self-assemble in water and carry drugs to different sites (Yadav et al., 2023). Liposomes are among the most used substances for drug delivery. In 2014, Hoekman et al. (2014) achieved better stability and analgesic effects in fentanyl-encapsulated liposomes by infusing them with integrin-targeting patterns for aerosol use (Hoekman et al., 2014). In a similar matter, polyester-based particles made of PLGA can be synthesized with ligands such as glycosylated heptapeptides for loperamide delivery, or lactoferrin and transferrin to better target pain centers (Nhàn et al., 2023; Forte et al., 2022). PEGylated particles infused with methylprednisolone or betamethasone proved efficient in increasing circulating time and decreasing toxicity (van Alem et al., 2021), while celecoxib-loaded agents coated with hyaluronate gel were more effective in pain control and articular protection than either of the substances separately when used in intra-articular administration (Wen et al., 2023). Postoperative pain management is a challenge for medical professionals, proving sometimes difficult to manage. In a clinical study by Gorfine et al., they observed the analgesic properties of bupivacaine long-release injections compared to placebo administration through wound infiltration. Liposomal nanoparticles had promising results in decreasing pain and opioid necessity over 3 days as opposed to the placebo (Gorfine et al., 2011). In another study by Lafont et al., liposomal-infused bupivacaine managed cancer-induced pain for as long as 11 h compared to approximately of 4h in classically administered medication (Lafont et al., 1996). Banshali et al., in the recent review discussed about the qualities of each type o nanoparticle used for pain management. More than that, The FDA has approved the commercialization of two extended-release morphine NDDSs (Nanoparticulate Drug Delivery Systems), Depodur and Avinza. Depodur utilizes the proprietary DepoFoam technology, a multivesicular liposomal delivery system that contains numerous non-concentric aqueous chambers filled with the drug. A single epidural injection of Depodur provides 48 h of analgesia. Avinza, which is taken orally, contains extended-release morphine capsules in proprietary beads made of ammonium-methacrylate copolymers. These beads are solubilized by gastrointestinal fluids, allowing the drug solution to diffuse out of the capsule and maintain therapeutic plasma levels for up to 24 h (Bhansali et al., 2021).

Nanocrystal−Iron Oxide Nanoparticles: they have multiple advantageous properties such as biodegradation, non-toxicity, and clearing pathway through the iron metabolism, with the special trait of magnetic actions that can be used in imaging and external guidance via magnetic fields (Dulińska-Litewka et al., 2019). There is limited research on the impact of iron oxide nanoparticles on pain management. However, a recent study by Ping-Ching Wu et al. demonstrated that local administration of iron oxide nanoparticles produced significant analgesic effects. This was achieved by reducing inflammatory cell infiltration and pro-inflammatory signaling, as well as scavenging free radicals in the microenvironment, in a mouse model of inflammatory pain (Wu et al., 2017).

Polymeric nanoparticle - Nanotubes: they can be charged with proteins, nucleic acids, or drugs and possess the ability to pass through cellular walls, showing high medication levels with low toxicity (Mondal et al., 2023). Quantum Dots: like their name, these nanoparticles have dimensions that range from 2 to 10 nm, and they consist of semiconductors with particular optical properties. Used in both diagnosis and treatment, these particles are utilized in fluorescent imaging as well as therapy delivery (Matea et al., 2017). The utilization of nanotechnology holds promise in alleviating the side effects of pain medications and curbing the development of tolerance, thus enhancing treatment efficacy. Various pain drugs can be encapsulated within diverse nanocarriers, for a spectrum of medical interventions (Zhu et al., 2023). For example, for migraine treatment, Girotra, cited by Bhanshali, encapsulated the GPCR agonists sumatriptan and zolmitriptan in various nanoparticles (Bhansali et al., 2021).

Polymeric nanoparticle−Nanofibers have been studied in relation to wound pain relief and infection prophylaxis. Chen et al. have concluded that electrospun PLGA or collagen membranes infused with antibiotics like vancomycin and gentamicin and analgesics such as lidocaine were successful in accelerating the healing process and reducing pain (Chen et al., 2012a; Chen et al., 2012b). Similarly, Wang et al. constructed a chitosan/PCL composite electrospun molecule loaded with rhodamine B and naproxen which has the property of controlled release, offering a new perspective on programmed drug delivery of more than one agent (Table 3; Wang et al., 2010; Saini et al., 2016; Mazurkiewicz-Pisarek et al., 2023; Shen et al., 2022; Prete et al., 2023; Jervis et al., 2020; John et al., 2022). One of the most useful advancements in pain management is the combination of nanotechnology and analgesic medication for rapid delivery and fast acting. Oral transmucosal fentanyl citrate (OTFC) is the first such technology and it is able to transport its active component, fentanyl, via mucosal pathways (Cerchione et al., 2020). High surface-volume ratios of nanoparticles infused with fentanyl increase drug-mucosal interaction and improve bioavailability in comparison to bigger molecules (Mangla et al., 2022). Payne and collaborators conducted a study about the long-term safety of OTFC, concluding that more than 90% of pain episodes were successfully managed with this treatment as well as seeing no decrease in efficiency after higher doses (Bahrami et al., 2020). In a 2013 study, Caraglia et al. (2013) worked with PEGylated liposomal nanoparticles for the delivery of zoledronic acid (ZOL) in neuropathic pain management. This substance consists of a ras-dependent Erk-mediated pathway inhibitor that blocks astrocyte conversion, but its effects are dampened by biodistribution limitations. The purpose of the study was to deliver ZOL in extra-bone tissue by enhancing the particle with PEGylated liposomes. Conclusions showed that it can now cross the blood-brain barrier and act directly on astrocytes, thus prolonging pain relief and lowering antalgic dosage (Mangla et al., 2022). Another type of nanomaterial used for coating is chitosan (CS). This additive was successfully used in modifying cannabinoid derivatives for better bioavailability and lower adverse effects. In a study by Durán-Lobato et al. (2015) they compared CS and PEGylated cannabinoid derivatives, concluding that CS ones performed better in interacting with Caco-2 cells, while PEG ones prevented adverse effects by having limited uptake in the THP1 cells (Durán-Lobato et al., 2015). Inflammatory response leads to high levels of TNF-α, which increases ROS production and sustains apoptosis. Thus, a new solution in the form of ROS scavengers began to be studied. Fullerol-enhanced particles showed promising results in managing inflammation by lowering ROS levels as well as upregulating antioxidative gene expression (Chuang et al., 2009). Another pathophysiological mechanism in chronic pain is the redistribution of substance P neurokinin 1 receptor (NK1R) from the cell membrane to the endosome, which is acidified and maintains pain. For this reason, pH-oriented nanoparticles were created to target acidic sites and enter NK1R-filled endosomes by endocytosis. This results in blockage of nociceptive transmission by inhibiting protein S activation and managing recurrent pain (Hung and Kuo, 2022).

**TABLE 3:** Nanofibers applications in wound pain management.

Material	Analgesic	Concurrent medication	Structure	Function	Reference
Poly (lactic acid)	Ibuprofen		Membrane	Antiinflammatory	Zhu et al., 2023
Chitosan polycaprolactone	Naproxen	Rhodamine B	Core-sheath	Analgesia/infection	Chen et al., 2012a
Poly (lactic-co-glycolic acid)	Lidocaine	Vancomycin	Sandwich	Analgesia/infection Accelerate wound healing	Chen et al., 2012b
Poly (epsilon-caprolactone)	Curcumin		Membrane	Antiinflammatory/ antioxidant	Wang et al., 2010
Phe-Phe	Naproxen		Hydrogel	Analgesia	Saini et al., 2016

### 3.1 Transdermal nanoparticle the new opportunities in pain management

There are several routes of administration for nanoparticle-infused drug delivery, but one that presents significant advantages is through microneedles. Bypassing degradation in the GI tract and pain upon administration, microneedles offer high bioavailability and increase patient compliance by self-administration and lower costs. There are four main types of particles utilized for transdermal delivery:

(1) nanocrystals, which consist of nanometer-sized pure drugs;
(2) lipid-based nanoparticles;
(3) polymeric nanoparticles and
(4) inorganic nanoparticles, which include silicas or metals (Jiang et al., 2022).

**FIGURE 4:**
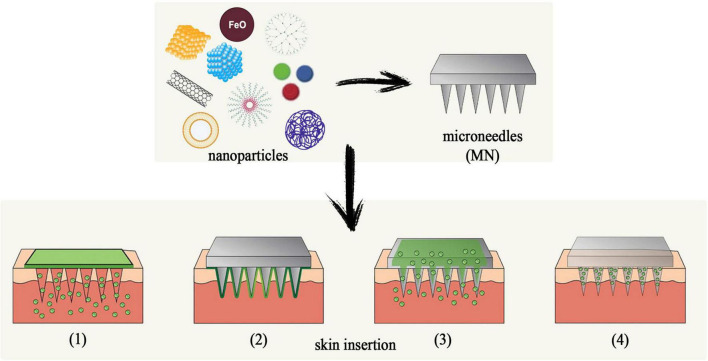
Nanoneedles–new opportunities in pain management.

Nanocrystals have been useful in issues with drugs that have low solubility step I because they are characterized by an increased surface area that offers better bioavailability and dissolution rates. Furthermore, the lack of coating on these particles has the advantage of high drug loading capacity, delivering treatments in pathologies that require high doses of therapeutics (Girard et al., 2023; Hsiang et al., 2022). Lipid-based nanoparticles mostly consist of liposomes, nanoemulsions, solid lipid nanoparticles (SLN), and nanostructured lipid carriers (Kashyap et al., 2023). Their main advantages are represented by their lipophilic properties which can increase the bioavailability of water-soluble agents, as well as their ability to fuse with the outermost layer of the epidermis to increase passage through the skin (Raut et al., 2021). In a study from by Khanna et al. (2020) a SLN model infused with nalbuphine was tested for intranasal use. Nalbuphine (NLB) is an opioid agonist/antagonist drug indicated in moderate to severe pain management. NLB-loaded SLNs had a loading capacity of approximately 26% and had an almost complete distribution rate of 8 h post-administration. The study also screened for toxicity levels in kidney cells, proving safe when administered nasally (Khanna et al., 2020). Polymeric nanoparticles prove superior in terms of biocompatibility, target recognition, cytotoxicity, and drug availability. The most used polymers are PLGA, poly (lactic acid) (PLA), PEG, chitosan, polyacrylates, albumin, gelatin, and alginates. However, two of them are the most utilized ones: PLGA on the hydrophobic spectrum because it has slow degradation properties and high biocompatibility, which can be useful in cases of controlled release need and PEG one the hydrophilic one for its flexibility and non-immunogenic characteristics (Jefferson and Linder, 2023; Guo et al., 2023; Rtimi et al., 2016; Shalom et al., 2017). A study conducted in 2023 by Zhang H. et al. (2023) evaluated sustained release esketamine delivery via PLGS and hyaluronic acid (HA) nanoparticle administration. This substance is a potent non-opioid medication efficient in neuropathic pain that has the limitations of a short half-life and psychiatric side effects. The purpose of the study was to decrease these shortcomings by enhancing esketamine with PLGA nanoparticles and HA hydrogel. Results showed continuous di-minishing of spinal nerve ligation-mediated hypersensitivity for up to two weeks with no pathophysiological alterations of the nerves. Furthermore, it was observed that neurons in the posterior root ganglions had inhibited excitability, and astrocyte activation was downregulated (Zhang H. et al., 2023). Finally, inorganic nanoparticles are the most advanced types because they offer a non-toxic, highly stable alternative to drug delivery. In the form of mesoporous silica nanoparticles (MSNs), superparamagnetic iron oxides (SPIONs), quantum dots, and metallic nanoparticles, these types of molecules have gained popularity in pain management and targeted therapy over recent years. MSNs have particular pore structure and size which can accommodate a large surface area that prevents enzyme degradation of the medication as well as the ability to be altered through post-modification with polymers or other molecules (Mihu et al., 2017). SPIONs have the unique properties of being controlled by external magnetic fields and serving as contrast agents in MRI diagnosis (Zhu et al., 2017). There are four ways to administer transdermal therapeutics, as seen in Figure 4: (1) topical application after MN penetration; (2) coated MNs; (3) dissolvable MNs, and (4) hollow MNs.

Puncturing the skin with solid MNs creates microscopic holes that allow nanoparticles to penetrate through the epidermis. This facilitates the delivery and deposit of the drug below the stratum corneum, achieving superior transdermal administration of nanoparticles (Peña-Juárez et al., 2022). Hollow MNs can pack larger quantities of medication that can be continuously infused through the inserted needles. One study by Mir et al. (2020) compared hollow MN administration of carvacrol with topical application of the substance, resulting in 8.5 times higher concentrations through the first route (Mir et al., 2020). Coated MNs contain substances underneath a barrier that dissolves upon skin insertion and allows the drug to dissolve at the site of the injection. However, this method cannot reach high doses of drug administered (Qi et al., 2023).

## 4 Nanotechnology and potential risk for children health

The limited availability of pediatric drugs often necessitates off-label prescriptions, commonly involving methods like splitting tablets or diluting injections. This practice can increase the risk of adverse drug reactions in children, either from overdosing or from therapeutic ineffectiveness due to subtherapeutic doses. In recent decades, the Food and Drug Administration (FDA) and the European Medicines Agency (EMEA) have encouraged the development of pediatric formulations to address the need for therapies tailored to specific weight and age requirements (Marques et al., 2022).

Factors influencing the development of pediatric drugs include pharmacotechnical challenges in creating manageable pharmaceutical forms, masking the taste of active ingredients, and selecting suitable inputs for different age groups. For patients under 2 years of age, liquid dosage forms are acceptable. Between 2 and 6 years, preferred forms include solutions, emulsions, suspensions, and effervescent medicines. For ages 6–12, orodispersible and chewable tablets are typically chosen. Ensuring the palatability of these medications is crucial for therapy compliance. Technologies proposed for taste masking include sweeteners and flavoring systems, bitter blockers and taste modifiers, modification of active pharmaceutical ingredients, solubility enhancement, and nanotechnology. Nanotechnology offers numerous benefits in the pharmaceutical field, such as higher therapeutic efficiency, targeted drug delivery, and low toxicity. These advantages make formulations incorporating nanotechnology an effective strategy for pediatric patients (Marques et al., 2022; Mfoafo et al., 2021).

The integration of nanotechnology in pediatric medicine has opened up new possibilities in fields such as pain management, oncopediatrics, pediatric pneumology, and the treatment of infectious or neurological diseases. However, this advancement also brings concerns about potential health risks. Recent studies highlight the impact of nanoparticles on children’s health, underscoring the necessity for thorough investigations into their safety profiles. Potential health hazards linked to nanosystems in pediatric nanomedicine encompass neurotoxicity, heightened metabolic strain, oxidative stress, inflammation, and, in the case of carbon nanoparticles, carcinogenicity (Omidian and Mfoafo, 2023). In their efforts to quantify nanoparticle exposure, researchers discovered that the physical characteristics of nanoparticles are closely linked to their intracellular dynamics and behavior. For instance, silver nanoparticles have been shown to trigger or worsen metal allergies, while amorphous silica nanoparticles can induce disseminated intravascular coagulation syndrome (Higashisaka, 2022).

It is critical to conduct a thorough evaluation of the potential dangers linked to nanotechnology (Omidian and Mfoafo, 2023). Numerous authors have evaluated this aspect, highlighting the potential effects of nanotechnology in the field of medicine, particularly in pediatrics, and emphasizing the value of exercising caution (He et al., 2022; Vishvakarma et al., 2023; Kulkarni et al., 2020). As an illustration, a research study examined the effects of engineered nanoparticles on the health of children, whereas two other studies (Mfoafo et al., 2021) concentrated on the neurotoxic properties of nanoparticles. Furthermore, additional research is necessary to determine the health effects of carbon nanoparticles. Research has demonstrated that nanoparticles are capable of causing oxidative stress and metabolic overload (Cai et al., 2021; Choudhary, 2022).

## 5 Conclusion

Pain is an endemic and escalating worldwide health concern that exerts substantial societal and economic ramifications on health systems, patients, and, by extension, society at large. Although contemporary treatment approaches embrace a diverse range of pharmacological and non-pharmacological methods, their efficacy is not universally consistent, particularly in pediatric patients, due to the intricate nature of pain and variations in clinical response among individuals. Furthermore, certain analgesics, including opioids, nonsteroidal anti-inflammatory drugs, and local anesthetics, cause a variety of undesirable side effects. Hence, the present advancements in research within this medical domain are predicated on the creation of prospective therapeutic interventions that can effectively mitigate existing constraints in pain management and confront numerous unfulfilled requirements. With careful consideration of the primary benefits that these systems provide, organic nanostructures and inorganic nanoparticles have been engineered to encapsulate various analgesics. The rapid evolution of nanomedicine is propelled by novel delivery strategies, technologies, treatment approaches, drug approvals, and even clinical setbacks of existing medications. The primary impediment to the advancement of pediatric nanomedicine at present is the inadequate establishment of clinical trial protocols.
